# A Plasma Exosomal Metabolic Profiling of Nonalcoholic Fatty Liver Disease Patients Complicated with Impaired Fasting Glucose

**DOI:** 10.5152/tjg.2024.22739

**Published:** 2024-02-01

**Authors:** Weiyun Jiang, Qiaofei Jin, Chunqing Li, Yunhao Xun

**Affiliations:** Department of Liver Disease, Hangzhou Sixth People’s Hospital/Xixi Hospital of Hangzhou Afflicted to Zhejiang University, Hangzhou, China

**Keywords:** Exosomes, extracellular vesicles, impaired fasting glucose, metabolomics, nonalcoholic fatty liver disease

## Abstract

**Background/Aims:**

Nonalcoholic fatty liver disease is considered as the hepatic manifestation of metabolic syndrome. Detection of circulating exosomes together with metabolomic analysis of their cargo would provide early signals for metabolic derangements and complications associated with nonalcoholic fatty liver disease. Therefore, this study profiled exosomal metabolome of patients with nonalcoholic fatty liver disease and impaired fasting glucose.

**Materials and Methods:**

Plasma exosomes were extracted from nonalcoholic fatty liver disease patients with or without impaired fasting glucose through differential ultracentrifugation. Their metabolite profiles were examined by ultrahigh-performance liquid chromatography–quadrupole time-of-flight mass spectrometry. Pathway analysis was carried out on platform MetaboAnalyst 4.0.

**Results:**

Thirty-nine patients were enrolled, including nonalcoholic fatty liver disease-alone group (n = 26) and age-and gender-comparable nonalcoholic fatty liver disease plus impaired fasting glucose group (n = 13). Although less than and different from their plasma counterparts, a total of 10 significantly differential exosomal metabolites were identified. Nonalcoholic fatty liver disease plus impaired fasting glucose group had higher concentrations of linoleic acid, palmitamide, stearamide, and oleamide, as well as a lower concentration of phosphatidylethanolamine [20:5(5Z,8Z,11Z,14Z,17Z)/20:5(5Z,8Z,11Z,14Z,17Z)]. Pathway analysis showed an obviously changed metabolism of linoleic acid.

**Conclusion:**

Metabolomic analysis of plasma exosomes revealed a distinct change in fatty acids and related pathways in nonalcoholic fatty liver disease patients with impaired fasting glucose. These preliminary results provide a metabolomic snapshot and basis for further investigation of exosome biology for these patients.

**Main Points**

- Nonalcoholic fatty liver disease (NAFLD) patients with impaired fasting glucose (IFG) are a representative entity to capture the subtle change in the early stage of metabolic derangement and hepatic injury.
- Metabolomic analysis of plasma exosomes revealed a distinct change in fatty acids and related pathways, higher concentrations of linoleic acid, palmitamide, stearamide, and oleamide, together with a lower concentration of phosphatidylethanolamine [20:5(5Z,8Z,11Z,14Z,17Z)/20:5(5Z,8Z,11Z,14Z,17Z)], in NAFLD patients with IFG.
- These preliminary results provide a metabolomic snapshot for further investigation of exosome biology in the pathogenesis of NAFLD in the era of terminology (N-/M-AFLD) changing.

## Introduction

Nonalcoholic fatty liver disease (NAFLD) is a leading cause of chronic liver disease, affecting a quarter of the adult population worldwide. The prevalence of NAFLD has increased over time, paralleling with the rising trend of obesity and type 2 diabetes mellitus (T2DM), and all of them causing significant health and economic burdens.^1^ Recently, the updated term metabolic dysfunction–associated fatty liver disease (MAFLD) of NAFLD has emerged in order to better denote the hepatic manifestation of a multisystem disorder that is heterogeneous in its underlying origin, progression, and outcomes.^2^ Metabolic dysfunction–associated fatty liver disease emphasizes again the importance of the metabolic aspect of this unique disease. Hence, an increasing demand to explore the metabolic pathophysiology of NAFLD is warranted.

Nonalcoholic fatty liver disease embraces the full pathological hepatic spectrum from simple steatosis to nonalcoholic steatohepatitis (NASH) and cirrhosis.^3^ The complex pathophysiology underlying the progression of NAFLD and its interplay with dysregulated glucose metabolism has been widely investigated but incompletely understood.^4^ In fact, an estimated 75% of NAFLD cases are complicated with prediabetes, either impaired fasting glucose (IFG), impaired glucose tolerance, or both.^5^ Impaired fasting glucose is the dominant type of prediabetes in patients with NAFLD, referring to an intermediate stage between normal glucose tolerance and overt T2DM.^6^ Emerging evidence suggests that IFG should also be viewed as a risk factor presaging the progression of NASH and fibrosis.^7,8^ The underlying pathophysiological disturbances of excess risk from IFG are believed to be the same as those from T2DM, with a central role played by insulin resistance.^9,10^

The progress of NAFLD and associated metabolic dysregulation is a complex process that relies on cross talk between metabolically active tissues.^11^ Extracellular vesicles (EVs), mainly including exosomes and ectosomes, are circulating nanovesicles secreted by most cell types and are compoed of bioactive cargoes that are horizontally transferred to targeted cells or tissues.^12^ A derailment in normal secretion and production of these metabolic mediators has been shown to contribute to the progression of NAFLD and related metabolic diseases.^13^ Studies have indicated the role of EVs as links between lipotoxicity, inflammation, angiogenesis, and fibrosis, all key events in the pathogenesis of NASH,^14^ and exosomes also play pivotal roles in the regulation of peripheral insulin sensitivity and whole-body glucose and lipid metabolism.^11^

To this end, a new trend is emerging to look for a wider and deeper panel of investigated exosome populations, pinpointing disease characteristics and clarifying the underlying mechanism for metabolic conditions.^15^ Exosomal metabolomics is a nascent field but one with great potential to reveal dynamic changes in the metabolism downstream of genetic and proteomic regulation.^16^ The technological revolution of metabolomics in separation and detection of small molecules, combined with rapid progress in bioinformatics, is making it possible to rapidly measure a large number of metabolites in a small amount of sample.^17^ Thus, detecting molecules enveloped in exosomes by nontargeted metabolomics technology is expected to provide a panoramic view of the metabolic shifts.

Until now, no literature has studied the metabolite species within plasma exosomes and how these profiles change during NAFLD progression. Herein, we conduct an exosomal metabolomics study on a group of NAFLD patients with IFG. It could be helpful to delineate the intrinsic nature of metabolic dysregulation in the pathogenesis of MAFLD, ultimately leading to the development of novel therapeutic approaches.

## Materials and Methods

### Participants

Subjects were recruited from our hospital from June 2019 to October 2019. Each participant’s demographic parameter and clinical characteristics were recorded. Ethical approval was obtained from the Institutional Ethics Committee of Xixi Hospital of Hangzhou, Zhejiang, People’s Republic of China (Approval number 2019, No. 12., Approval date: 01/10/2019). All participants provided written informed consent prior to the study.

Nonalcoholic fatty liver disease is defined by excess steatosis in ultrasound with the absence of other causes such as alcohol abuse, viral infection, drug-induced liver injury, autoimmune liver disorders, or Wilson’s disease.^18^ Impaired fasting glucose is defined as an IFG level (5.6-6.9 mmol/L), based on the standard formulated by the American Diabetes Association in 2018.^19^ Exclusion criteria are as follows: patients with hepatitis B or C virus or toxic liver disease or autoimmune liver disease or Wilson’s disease, overt T2DM, on treatment of IFG, any other severe systemic or mental diseases, and females of childbearing age who were pregnant or lactating.

### Sample Collection and Preparation

Blood sample collection was strictly performed in accordance with exosome preparation recommendations.^20^ Peripheral blood (5 mL for each) were collected into heparin-containing tubes from overnight fasting subjects (≥8 hours since last meal) and centrifuged for 3000r × 10 minutes at room temperature. The supernatant was split into 2 fractions with labeled tubes and stored at −80°C, used for plasma and exosomal metabolomics profiling, respectively. The following analysis was performed on a commercial platform at Lc-Bio Technologies Co., Ltd (Hangzhou, China).

### Exosomes Isolation and Validation

At present, each EV isolation method has its own advantages and disadvantages; ultracentrifugation is still the most commonly used one up to now.^21,22^ Limited by local technical resources and for a convenient comparison to historical metabolomics studies, we chose ultracentrifugation to prepare EV samples followed by serial methods to characterize the products qualitatively and quantitatively.

After being thawed at 37°C, samples were differentially centrifuged at 3000 *g *for 30 minutes and 12 000 *g* for 45 minutes. The supernatant was then filtered (0.45 μm) and recentrifuged at 110 000 *g* for 70 minutes at 4°C. The pellets containing exosomes were resuspended in phosphate-buffered saline (PBS) and centrifuged at 110 000 *g* for 70 minutes again. Resultant pellets were resuspended in PBS buffer and then stored at 4°C for less than 3 days for further experiments. A mixed sample (70 μL) was used for the following characterization of the exosomes.

### Transmission Electron Microscopy and NanoSight

Isolated exosomes were deposited on a copper grid, negatively stained using phosphotungstic acid, then were blotted and air-dried. A transmission electron microscope (FEI Tecnai Spirit T12) was used for imaging. The size distribution and particle concentrations of exosomes were determined by nanoparticle tracking analysis with NanoFCM (Fuliu Biology, Flow NanoAnalyzer).

### High-Sensitivity Flow cytometry (NanoFCM)

Isolated exosomes were divided into 3 equal parts, labeling with fluorescein isothiocyanate (FITC) Mouse Anti-Human CD9 (BD, 555371, Shanghai, China), FITC Mouse Anti-Human CD81 (BD, 551108), and FITC Mouse IgG (BioLegend, 400108, Shanghai, China) respectively. The correspondingly FITC signals were examined following manufacturer’s instructions.

### Western Blotting

Exosomal protein was extracted by resuspending the exosomes in radio immuno precipitation assay (RIPA) lysis and extraction buffer as per protocol. About 12 μg of resultant protein was separated on a 10% sodium dodecyl sulfate - polyacrylamide gel electrophoresis (SDS-PAGE) gel, transferred to a polyvinylidene fluoride membrane, followed by immunodetection of calnexin (Mouse Anti-Human, Abcam, ab22595, Shanghai, China), as negative control for exosomes, with chemiluminescent detection.

### Metabolite Extraction

The exosomal samples prepared earlier were thawed on ice, extracted with 120 μL of precooled 50% methanol, vortexed for 1 minute, and kept at room temperature for 10 minutes. The mixtures were stored overnight at –20°C for deproteinization. After centrifugation at 4000 *g* for 20 minutes, the resulting supernatants were transferred into 96-well plates for ultrahigh-performance liquid chromatography–time-of-flight tandem mass spectrometry (UHPLC-Q-TOF-MS/MS) analysis. In addition, a small fraction of supernatant from each sample was combined into a pool as control (pooled quality control [QC]).

### Ultrahigh-Performance Liquid Chromatography–Quadrupole Time-of-Flight Mass Spectrometry Assay

Chromatographic separations were performed on an UHPLC system (SCIEX, Shanghai, China). An ACQUITY UPLC T3 column (100 mm × 2.1 mm, 1.8 μm, Waters, Shanghai, China) was used for the reversed-phase separation. The mobile phase consisted of solvent A (water, 0.1% formic acid) and solvent B (acetonitrile, 0.1% formic acid). Gradient elution conditions were set as follows: 0-0.5 minutes, 5% B; 0.5-7 minutes, from 5% to 100% B; 7-8 minutes, 100% B; 8-8.1 minutes, from 100% to 5% B; 8.1-10 minutes, 5% B. The column flow rate was 0.4 mL/min, injection volume was 4 μL, and column oven was maintained at 35°C.

Metabolites eluted from the column were detected using a high-resolution tandem mass spectrometer TripleTOF 5600 plus (SCIEX), operated in both positive and negative ion modes. The curtain gas was set as 30 psi, ion source gas 1 was set as 60 psi, ion source gas 2 was set as 60 psi, and an interface heater temperature was 650°C. The ion spray voltage floating of positive/negative ion mode were +5.0/−4.5 kV respectively. The mass spectrometry data were acquired in information-dependent acquisition mode. The TOF mass range was from 60 to 1200 Da. The survey scans were collected in 150 milliseconds and up to 12 product ion scans were acquired if exceeding a threshold of 100 counts per second (counts/s) and with a 1+(−) charge-state for positive (negative) ion mode. Total cycle time was 0.56 seconds. Four time bins were summed for each scan at a pulser frequency value of 11 kHz through monitoring of the 40 GHz multichannel thermal conductivity detector with 4-channel detection. Dynamic exclusion was set for 4 seconds. The mass accuracy was calibrated every 20 samples during the acquisition. Furthermore, in order to evaluate the stability of the LC-MS, QC samples were analyzed between every 10 samples during the entire analytical sequence.

### Data Pretreatment and Data Analysis

The acquired LC-MS raw data were converted into mzXML format and then processed by the XCMS, CAMERA, and metaX toolbox implemented with the R software. After pretreatment, including peak picking, peak grouping, retention time correction, second peak grouping, and annotation of isotopes and adducts, the resulting 3-dimensional matrix containing arbitrarily assigned peak indices (retention time [RT] – molecular weight [*m*/*z*] pairs), sample names, and ion intensity information was generated. Secondary metabolites with detectable features (defined by a pair of *m*/*z* and RT) were annotated with the aid of in-house reference standard library of Lc-Bio Technologies and web-based resources, that is, the Human Metabolome Database (HMDB) and Kyoto Encyclopedia of Genes and Genomes (KEGG) database. The resulting normalized data were analyzed by multivariate statistical analysis using metaX, including unsupervised principal component analysis (PCA) and partial least squares discriminant analysis (PLS-DA). Selected results were visualized by hierarchical heat map. The differentially expressed metabolites with HMDB accession numbers were subjected to pathway analysis using the KEGG database on platform MetaboAnalyst 4.0 (http://www.metaboanalyst.ca).

Descriptive statistics of baseline demographic and clinical variables were performed on Statistical Package for the Social Sciences (SPSS) 19.0 (SPSS Inc., Chicago, Ill, USA) and compared using a Mann–Whitney U-test for continuous variables or Fisher’s exact test for categorical variables. All reported values are presented as median and quartiles (P25, P75). *P* < .05 was considered as statistically significant.

## Results

### Characteristics of the Study Participants

A total of 39 patients were enrolled in our study, including NAFLD-alone group (n = 26) and NAFLD plus IFG group (n = 13). No demographic differences between groups were observed for age and sex distribution. Besides the level of fasting blood glucose and glycosylated hemoglobin, NAFLD patients with IFG also had a higher concentration of γ-glutamyl transferase (GGT) and alkaline phosphatase than NAFLD-alone individuals (*P *< .05 for all). And none of the remaining parameters, including fasting insulin, liver enzymes and lipid profile, showed significant differences. Baseline demographic and clinical characteristics are detailed in Table 1.

### Morphological Characterization and Identification of Exosomes

**Figure 1.**
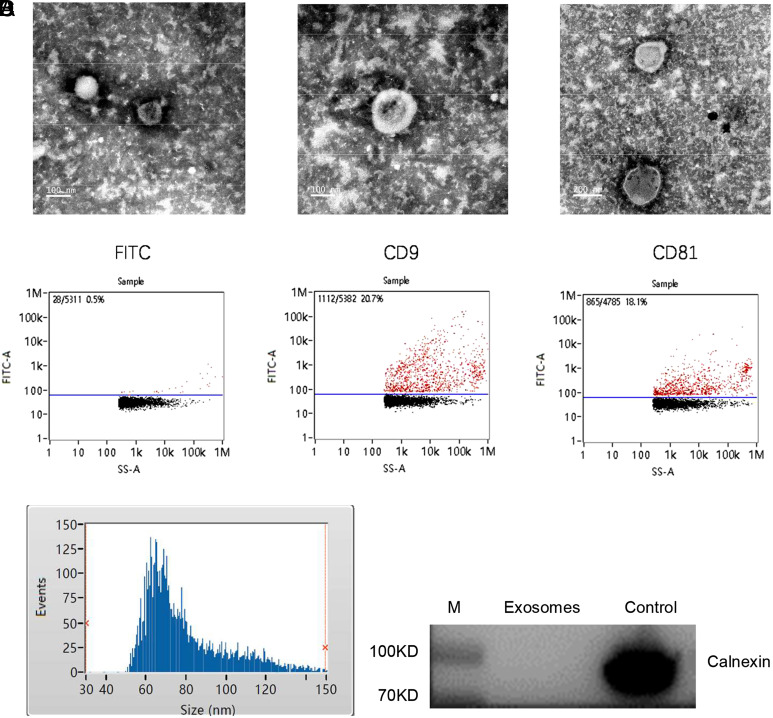
Validation of exosomal sample quality. (A) Representative TEM image of isolated exosomes. (B) Flow cytometry of exosomes detected by FITC Mouse Anti-Human CD9, CD81, and IgG. (C) Size distribution of exosomes on NanoSight. (D) Western blotting of the isolated exosomes confirmed the absence of endoplasmic reticulum protein calnexin. FITC, fluorescein isothiocyanate; TEM, transmission electron microscopy.

Quality of exosomes recovered from human plasma is demonstrated in Figure 1. Transmission electron microscopy revealed the presence of spherical nanometer-sized microvesicles (Figure 1A). NanoFCM showed the positive signals of 2 tetraspanins, CD9 and CD81 (Figure 1B). Physical characteristics of the isolated exosomes by NTA analysis revealed a size distribution of 79.01 ± 20.24 (nm) (Figure 1C). Western blotting of exosomes showed the absence of the endoplasmic reticulum marker calnexin (Figure 1D). These results showed that circulating exosomes (or small EVs under the more stringent criteria endorsed by The International Society for Extracellular Vesicles [ISEV]) were efficiently isolated.

**Supplementary Figure 1.**
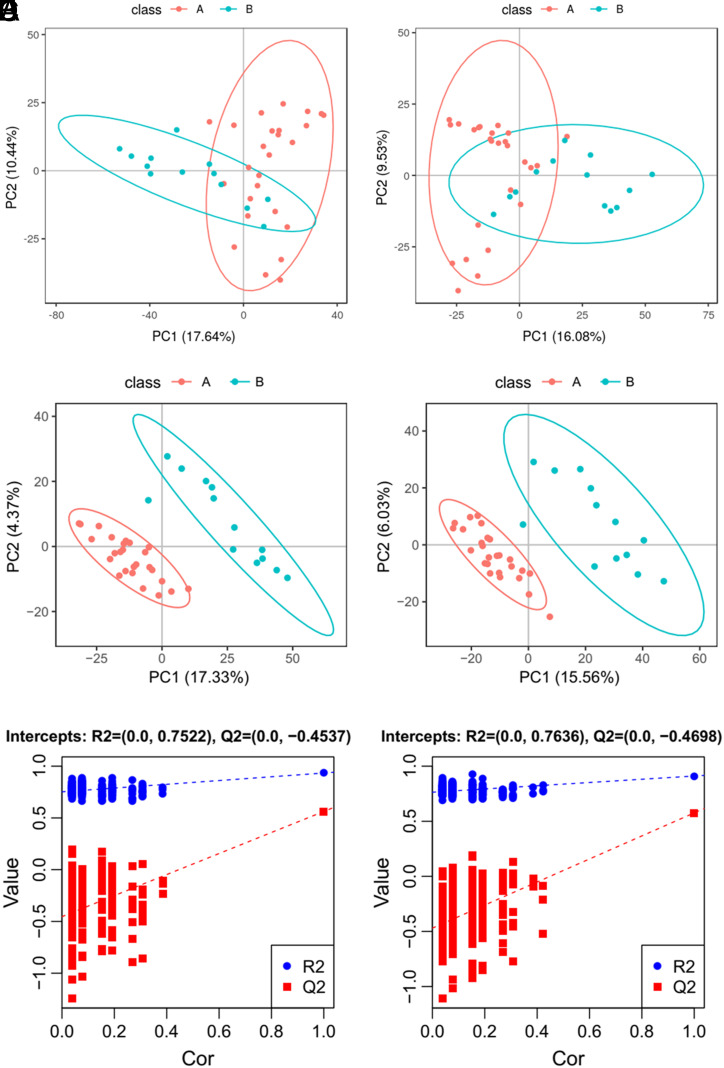
Metabolites multivariate analysis (A: NAFLD plus IFG; B: NAFLD alone group). (A, B) The score plots of PCA showed separation between two groups. The first and second components explained 17.64% and 16.08% of variations, respectively. (C, D) The score plots of PLS-DA showed the discrimination between the sample groups. (E, F) the corresponding validation plots based on 200 times permutation tests demonstrated the robustness of the PLS-DA models.

Taking into account of the possible contamination of lipoproteins and other EVs pertaining to the method of ultracentrifugation, a parallel analysis of plasma metabolomics was conducted as a trade-off. The following text focuses on the result of exosomal metabolomics, along with plasma nontargeted metabolomics profiling described in the supplementary material (multivariate analysis of plasma metabolites is shown in Supplementary Figure 1).

### Nontargeted Metabolomics Analysis

In positive mode, approximately 4596 spectral features of exosomal samples were obtained after deleting the repeated features, and 260 secondary metabolites in line with our in-house standardized reference library were annotated subsequently. As for the negative mode, 3063 spectral features and 74 secondary metabolites were acquired.

**Figure 2.**
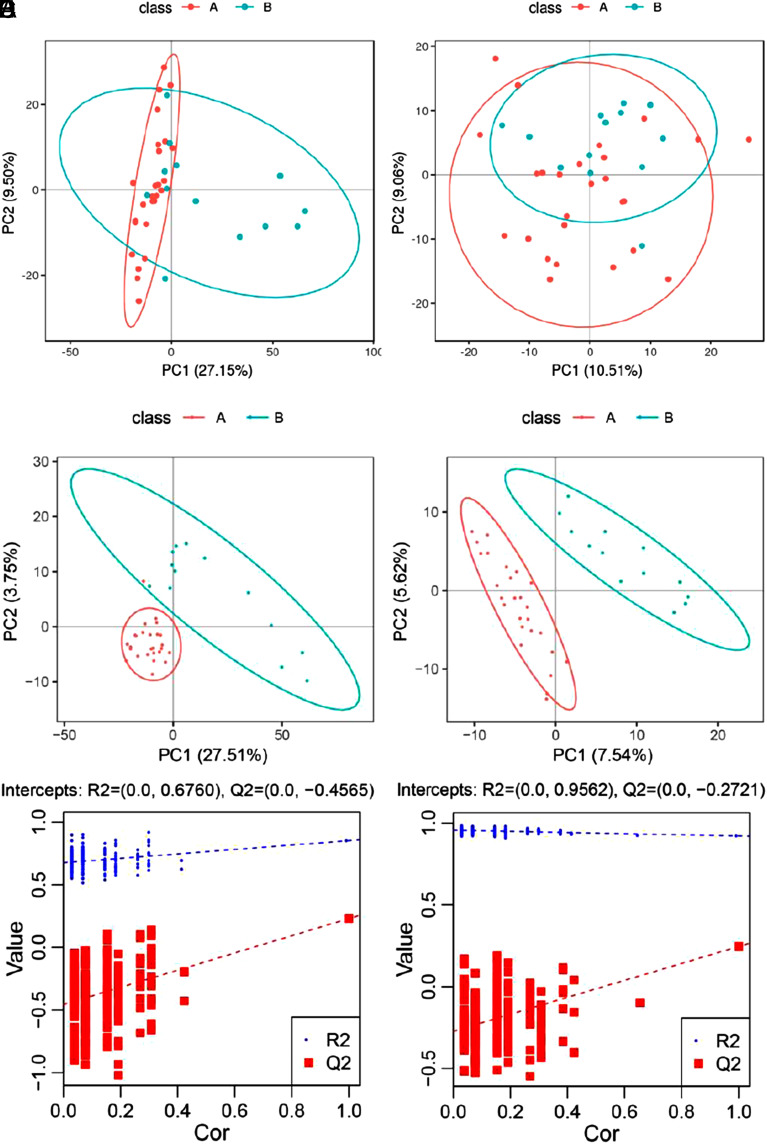
Metabolites multivariate analysis [(A) NAFLD plus IFG; (B) NAFLD-alone group]. (A, B) The score plots of PCA showed separation between 2 groups. The first and second components explained 27.15% and 9.50% of variations, respectively. (C, D) The score plots of PLS-DA showed the discrimination between the sample groups. (E, F) The corresponding validation plots based on 200 times permutation tests demonstrated the robustness of the PLS-DA models. IFG, impaired fasting glucose; NAFLD, nonalcoholic fatty liver disease; PCA, principal component analysis; PLS-DA, partial least squares discriminant analysis.

**Figure 3.**
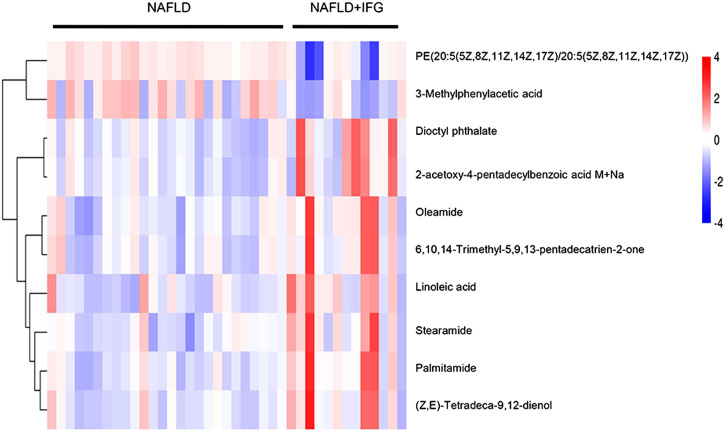
Heat map representation of the exosomal metabolomic profiles. Each data point corresponds to the relative ion abundance of a given metabolite (vertical axis) in an individual. The hierarchical clustering is based on 2 groups. Shades of blue and red represent concentration decrease or increase of a metabolite, respectively.

The PCA and PLS-DA models were conducted to visualize the metabolic differences between the 2 groups. However, examination of the PCA score plots (Figure 2A and 2B) provided an unsatisfactory separation of data between the 2 groups. The PLS-DA plots (Figure 2C and 2D) separated metabolites of the 2 groups clearly, with an *R* ^2^ and *Q* ^2^ (cum) of 0.852 and 0.230 in ESI+, 0.921 and 0.250 in ESI− respectively. The high value of *R* ^2^ indicated a good ability of explaining the metabolic variations between the 2 groups. The value of *Q* ^2^ is also acceptable. The validity and potential over-fitting of the PLS-DA model were checked by performing two hundred permutation tests (Figure 2E and 2F). As expected, the *R* ^2^ and *Q* ^2^ values in positive mode were lower than the original ones. However, the *R* ^2^ value in the negative mode was a little higher than the original one, indicating an over-fitting phenomenon. Thus, we kept the results of the positive mode only for further analysis. Additionally, heat maps were drawn by concentrations of differential metabolite to visualize their different distribution of the 2 groups (Figure 3).

### Differential Metabolites Identification

**Figure 4.**
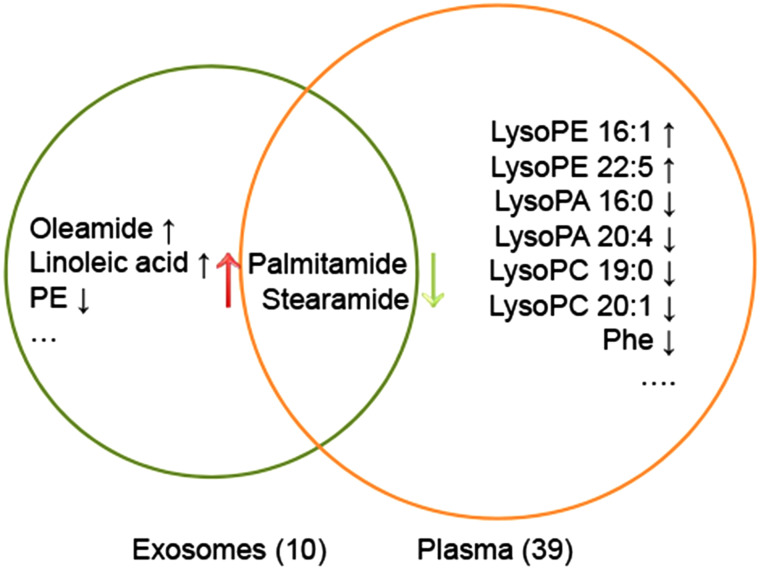
Venn diagram of the identified differential metabolites from circulating exosomes and their matched plasma samples in NAFLD patients with IFG. Amounts of exosomal metabolites identified were obviously less than their plasma counterparts. Palmitamide and stearamide were both detected in 2 batches of samples, while upregulated in exosomes and downregulated in plasma in NAFLD plus IFG patients. ↑, upregulated; ↓, downregulated; IFG, impaired fasting glucose; NAFLD, nonalcoholic fatty liver disease; PCA, principal component analysis; PLS-DA, partial least squares discriminant analysis.

Differential metabolites were screened according to the following criteria: (i) a *P*-value < .05, (ii) a fold change of ≥2 or ≤0.5, (iii) a variable importance in the projection value >1.0. A total of 10 metabolites from exosomes were identified as different in relative abundance between the 2 groups (Table 2). Plasma differential metabolites are shown in Supplementary Table 1. Palmitamide and stearamide were both detected in 2 batches of samples, while they were upregulated in exosomes and downregulated in plasma in NAFLD plus IFG patients. A detailed comparison between exosomal and plasma metabolomics, in terms of metabolites of distinction in NAFLD patients with IFG, is illustrated in Figure 4.

**Figure 5.**
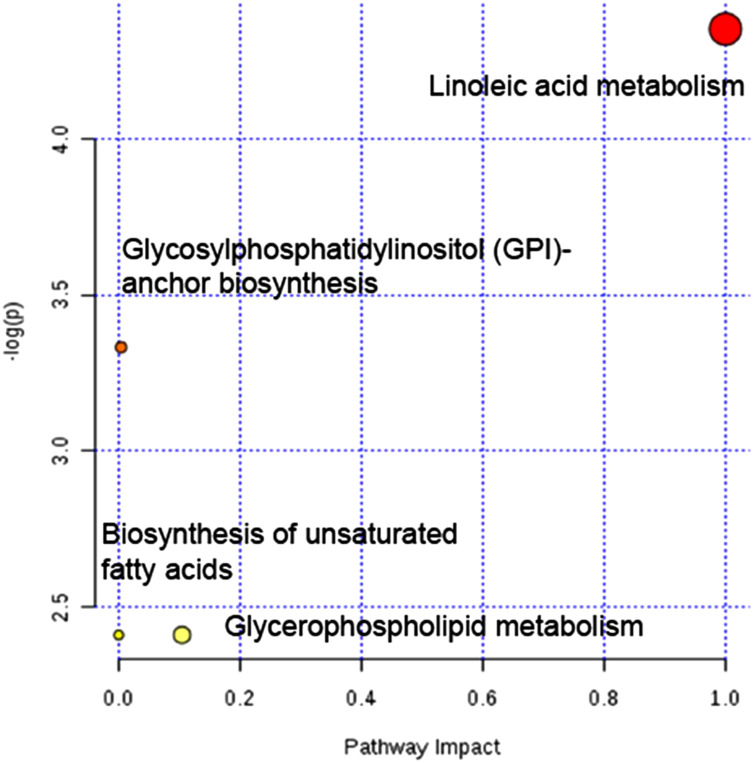
Summary of metabolic pathway analysis by MetaboAnalyst 4.0. Linoleic acid metabolism has been identified as the most significantly altered pathway.

According to the impact value calculated from pathway topology analysis, linoleic acid metabolism has been identified as the most significantly altered one (Figure 5).

## Discussion

Nonalcoholic fatty liver disease, or referred to as MAFLD currently, is a heterogeneous entity, and detailed metabolic stratification will probably yield more informative and meaningful subtyping.^2^ All but one (in NAFLD-alone group) patients enrolled in our study fulfilled the criteria of MAFLD; thus, the results presented here are appropriate in the era of terminology changing.

Although compromised by the small sample size, patients between the 2 groups were well comparable in gender and age. As a state of prediabetes and a typical marker for increased hepatic endogenous glucose production in NAFLD, the emergence of IFG indicates a slightly advanced phenotype. As expected, no obvious differences between NAFLD-alone group and NAFLD plus IFG group in terms of routine biochemical parameters such as liver enzymes and lipid parameters were observed. Elevation of GGT is a sensitive biomarker for hepatic inflammation and fibrosis^23^ and an early signal to inform the development of prediabetes and T2DM.^24^ Thus, NAFLD patients with IFG turn out to be a representative entity to capture the subtle change in the early stage of metabolic derangement and hepatic injury.

A clear understanding of disease phenotypes of NAFLD has been informed by advance in “omics” technology.^25^ Approximately 300-400 circulating metabolites have been reported through metabolomics in the literature, with significant correlations to elevated plasma glucose levels, insulin resistance, and so on.^26^ This study similarly demonstrated a lot of plasma differential metabolites related to dysglycemia state in NAFLD patients, as shown in the supplementary material.

There is a lack of knowledge of the pool of exosomal metabolites. Herein, we addressed the changed metabolic contents of peripheral exosomes in NAFLD patients complicated with IFG, in order to capture the early metabolic derangement underlying the deterioration of NAFLD. And due to the technical difficulty in characterizing plasma exosome type, we choose to explore the qualitative and quantitative differences in the harvested lipid metabolites but not the total concentration of exosomes between the 2 groups.

The real-time data obtained through metabolomics revealed an abundance of metabolites enveloped in exosomes in patients with NAFLD plus IFG, especially a higher concentration of several distinct fatty acids (palmitamide, oleamide, stearamide, and linoleic acid) as well as a decreased tendency in phosphatidylethanolamine [PE; 20:5(5Z,8Z,11Z,14Z,17Z)/20:5(5Z,8Z,11Z,14Z,17Z)]. These metabolites were complementary to their plasma counterparts and partially in line with the reported exosomal lipids.^27^ We acknowledge that the recovered exosomes by us might be unfortunately contaminated by lipoproteins to a certain extent, and the explanation of the results needs some caution. But we have to stress that neither of the currently available EV isolation methods is able to provide purely plasma origin EV samples.^28^ Thus our results would be even meaningful though less rigor somewhat.

Each individual fatty acid has its own specific actions. Palmitamide, oleamide, and stearamide belong to the primary fatty acid amides (PFAMs), a newer group of lipids that have been identified as bioactive lipid signaling molecules.^29^ Presumably, functions of exosomal metabolites might be different from that of the same metabolites excreted in a soluble form.^30^ The levels of palmitamide and stearamide were intriguingly upregulated in exosomes while downregulated in plasma, suggesting a potential role of PFAMs. And mechanistic studies are warranted due to the lack of information about their metabolism or localization in vivo.

This study found a significantly disturbed linoleic acid pathway in NAFLD patients accompanied by IFG. A majority of studies on linoleic acid and its derivatives, such as arachidonic acid, 9 and 13 hydroxyoctadecanoic acids (9-HODE and 13-HODE), showed a direct or indirect link with inflammation and metabolic diseases.^31-33^ Linoleic acid could produce 9-HODE through the nonenzymatic oxidation pathway. It has been reported that patients with NASH had significantly higher levels of 9-HODE and 13-HODE than simple steatosis patients.^34^ And linoleic acid-derived epoxides have been found increased in diabetic patients.^35^ Arachidonic acid, another product of the linoleic acid metabolic pathway, can be further metabolized to cyclooxygenase-2 (COX-2) through the cyclooxygenase or lipoxygenase; COX-2 then yields pro-inflammatory products such as prostaglandin E2 and thromboxane, both of which have been implicated in the pathogenesis of metabolic inflammation.^36^ Thus, we hypothesized that linoleic acid carried within exosomes has a potential role of pro-inflammation for target receptors and contributes to the abnormal metabolism in NAFLD patients with IFG.

The level of PE [20:5(5Z,8Z,11Z,14Z,17Z)/20:5(5Z,8Z,11Z,14Z,17Z)] was downregulated in NAFLD and IFG patients. This decreased PE, though with a complex origin, was well in accordance with the results of a mechanistic mouse study, that is, a high-fat diet dramatically changes the lipid profile of intestinal epithelial exosomes from predominantly PE to phosphatidylcholine (PC), thus resulting in inhibition of the insulin response via binding of exosomal PC to aryl hydrocarbon receptor expressed in hepatocytes and suppression of genes essential for activation of the insulin pathway.^37^ As a major component of biological membranes localizing in mitochondrial inner membranes, the altered content of PE may have even more potential functions.^38,39^

Several limitations have to be declared. First, the separation of pure EVs from human blood represents a tough challenge, so does the unambiguous identification of each metabolite in metabolomics. Thus, our results are only regarded as exploratory and inconclusive until be reproduced by more sophisticated approaches. Hopefully, EV characterization seems not insurmountable with the ongoing progression of technologies in the near future. Second, histological validation of the clinical phenotype was absent, thus weakening the strength for metabolome interpretation in hepatic inflammation and fibrosis somewhat. Third, the sample size may not be large enough to detect all disease-associated metabolite changes in plasma exosomes. Finally, given its cross-sectional design, our study does not allow inferring the cause–effect relationship between exosome metabolic profiles and IFG among NAFLD patients. Therefore, further investigations with a larger sample size, prospective design, and advanced technology are needed to confirm our findings.

Taken as a whole, our preliminary study indicates a distinct pattern of fatty acid profiles in NAFLD patients complicated with IFG, and dysregulated linoleic acid metabolism and decreased exosomal PE are tightly associated with this type of prediabetes as well as a possible severe hepatic condition. This metabolomic snapshot would be helpful for future investigations of exosome biology for these patients. However, it is still the subject of investigation whether these lipids serve only as predictors or also contribute toward the pathogenesis of NAFLD/MAFLD.

## Figures and Tables

**Table 1.** Demographic and Clinical Characteristics of NAFLD Patients

Group	NAFLD plus IFG (n = 13)	NAFLD Alone (n = 26)	*t*/*Z *Value	*P*
Gender				
Male	7 (53.8%)	22 (84.6%)	/	.056^*^
Female	6 (46.2%)	4 (15.4%)		
Age (years)	40 (22-51.5)	26 (23-31.5)	−1.463	.143
BMI (kg/m^2^)	30.95 (25.43-34.18)	27.90 (26.65-30.25)	−0.909	.363
FBG (mmol/L)	6.35 (6.14-6.61)	5.20 (4.84-5.41)	−5.036	<.0001
FINS (mU/L)	14.10 (12.52-15.69)	12.0 (7.70-16.96)	−0.969	.332
ALT (U/L)	111 (64-136)	74.5 (41.5-118.5)	−1.102	.270
AST (U/L)	57 (45.5-103.5)	50.5 (29.5-65.25)	−1.805	.071
ALP (U/L)	108.0 (90.5-119.0)	88 (66.0-106.5)	−2.011	.044
GGT (U/L)	93 (48-134.5)	50.5 (33.75-68.25)	−2.414	.016
HbA1c (%)	6.20 (5.90-6.65)	5.30 (5.00-5.40)	−4.445	<.0001
TC (mmol/L)	5.76 (4.88-6.47)	5.19 (4.32-6.07)	−1.281	.200
TG (mmol/L)	2.22 (1.35-3.28)	1.60 (1.27-2.44)	−0.834	.404
UA (μmol/L)	453.5 (379.3-532.63)	455.85 (417.38-543.2)	−0.6000	.510
Ferritin (μg/L)	370.4 (235.8-474.4)	251.5 (167.2-420.7)	−1.344	.179

ALP, alkaline phosphatase; ALT, alanine aminotransferase; AST, aspartate aminotransferase; BMI, body mass index; FBG, fasting blood glucose; FINS, fasting insulin; GGT, γ-glutamyl transferase; HbA1c, glycosylated hemoglobin; IFG, impaired fasting glucose; NAFLD, nonalcoholic fatty liver disease; TC, total cholesterol; TG, triglycerides; UA, uric acid.

^*^ Fisher’s exact test.

**Table 2.** Exosomal Differential Metabolites Between NAFLD plus IFG Group and NAFLD-Alone Group

Class	Fatty Acid	Fatty Acid	Fatty Acid	Fatty Acid	Unknown	Unknown	Glycerophospholipid	Unknown	Unknown	Unknown
*P*	.012	.013	.019	.011	.004	.044	.008	.027	.010	.017
Ratio	4.899	6.101	2.680	2.108	0.443	6.354	0.492	2.956	2.104	2.082
VIP	1.783	1.848	1.485	1.405	1.524	1.730	2.529	1.513	2.362	2.106
Trend	↑	↑	↑	↑	↓	↑	↓	↑	↑	↑
Compound	Palmitamide	Oleamide	Stearamide	Linoleic acid	3-Methylphenylacetic acid	6,10,14-Trimethyl-5,9,13-pentadecatrien-2-one	PE(20:5(5Z,8Z,11Z,14Z,17Z)/20:5(5Z,8Z,11Z,14Z,17Z))	(Z,E)-Tetradeca-9,12-dienol	Dioctyl phthalate	2-acetoxy-4-pentadecylbenzoic acid M+Na
RT	5.7925	6.0113	6.7351	6.0627	2.9725	6.06	2.4559	4.8293	391.28357	413.26541
*m*/*z*	256.2629	282.27885	284.29435	281.24702	151.0748	263.23659	784.48921	228.2316	391.28357	413.26541
NO.	1	2	3	4	5	6	7	8	9	10

IFG, impaired fasting glucose; *m*/*z*, mass–charge ratio; NAFLD, nonalcoholic fatty liver disease; RT, retention time; VIP, variable importance in the projection; PE, phosphatidylethanolamine.

**Supplementary Table 1.** Plasma Differential Metabolites between NAFLD Plus IFG Group and NAFLD Alone Group

No.	m/z	RT	Compound	Trend	VIP	Ratio	*P* value	Class
1	256.263	5.795267	Palmitamide	↓	1.946	0.218	0.012	Fatty acids
2	284.2947	6.736992	Stearamide	↓	2.152	0.174	0.006	Fatty acids
3	295.263	7.474375	Linoleic acid methyl ester	↓	2.242	0.411	0.000	Fatty acids
4	450.2634	3.579025	LysoPE 16:1	↑	2.089	2.047	0.000	Glycerophospholipids
5	528.2984	3.728583	LysoPE 22:5	↑	2.138	2.116	0.000	Glycerophospholipids
6	409.2203	2.75095	LysoPA 16:0	↓	2.411	0.480	0.002	Glycerophospholipids
7	457.2373	5.232633	LysoPA 20:4	↓	2.613	0.478	0.002	Glycerophospholipids
8	481.2372	5.088525	LysoPA 22:6	↓	2.290	0.488	0.003	Glycerophospholipids
9	521.338	4.947133	PA 23:0; PA(5:0/18:0)	↓	2.392	0.436	0.002	Glycerophospholipids
10	538.3864	5.532775	LysoPC 19:0	↓	2.056	0.488	0.000	Glycerophospholipids
11	550.3862	5.178317	LysoPC 20:1	↓	2.147	0.490	0.000	Glycerophospholipids
12	464.3029	2.8421	3a,7b,12a-Trihydroxyoxocholanyl-Glycine	↑	2.079	2.186	0.002	Sterol Lipids
13	448.3081	3.158425	Glycoursodeoxycholic acid	↑	1.765	2.212	0.022	Sterol Lipids
14	395.1897	3.379875	Pregnenolone sulfate	↓	2.622	0.389	0.000	Sterol Lipids
15	144.1013	0.957225	Stachydrine	↑	2.336	2.937	0.001	Carboxylic acids and derivatives
16	247.1285	2.23835	Aspartyl-Leucine	↑	2.404	0.474	0.002	Carboxylic acids and derivatives
17	313.1543	2.398817	Phe-Phe	↓	2.983	0.185	0.000	Carboxylic acids and derivatives
18	263.2367	5.60465	6,10,14-Trimethyl-5,9,13-pentadecatrien-2-one	↓	1.555	0.490	0.001	Prenol lipids
19	287.2367	5.941433	13-cis Retinol	↓	2.263	0.471	0.000	Prenol lipids
20	310.3097	6.87395	Geranylcitronellol	↓	1.650	0.297	0.002	Prenol lipids
21	593.4751	7.432517	SM 26:0;SM(d14:0/12:0)	↓	1.732	0.482	0.001	Sphingolipids
22	142.0475	2.319883	2-Aminonaphthalene	↑	2.372	3.813	0.007	Naphthalenes
23	144.0631	2.329267	(S)-5-Amino-3-oxohexanoate	↑	3.472	6.141	0.000	Keto acids and derivatives
24	153.0637	1.876333	Biphenyl	↑	2.802	3.804	0.001	Benzene and substituted derivatives
25	158.0789	2.371742	1-Piperidinecarboxaldehyde	↑	2.655	4.049	0.004	Piperidines
26	149.0441	2.063133	p-Coumaraldehyde	↑	2.206	2.640	0.000	Unknown
27	160.1325	0.996383	2-Octenoic acid, (E)-	↑	1.888	2.022	0.001	Unknown
28	255.1222	2.76665	Benzamide, N-1H-pyrrolo[2,3-c]pyridin-5-yl-	↑	1.816	2.107	0.009	Unknown
29	286.1433	3.385292	Piperin	↓	1.930	0.463	0.023	Unknown
30	319.263	7.218383	omega.-3 Arachidonic acid methyl ester	↓	2.454	0.406	0.000	Unknown
31	321.2784	7.74315	Dihomo-.gamma.-linolenic acid methyl ester	↓	2.440	0.458	0.006	Unknown
32	343.2627	6.966617	Docosahexaenoic acid methyl ester	↓	2.183	0.471	0.001	Unknown
33	375.2022	2.25085	Furanylfentanyl	↓	2.369	0.385	0.000	Unknown
34	506.3604	4.961008	N-Octanoylsphingosine-1-phosphate	↓	2.791	0.463	0.000	Unknown
35	508.3756	4.590108	1-(1Z-Octadecenyl)-sn-glycero-3-phosphocholine	↓	2.403	0.471	0.000	Unknown
36	510.3911	5.436925	1-O-Octadecyl-sn-glyceryl-3-phosphorylcholine	↓	2.496	0.415	0.000	Unknown
37	573.3137	2.2535	Bradykinin (1-5)	↓	1.364	0.196	0.048	Unknown
38	384.0857	1.878092	Tazarotenic acid sulfoxide	↑	2.479	2.720	0.001	Unknown
39	397.2053	3.01855	(3.beta.)-Allopregnanolone sulfate	↓	2.600	0.398	0.000	Unknown

m/z: mass charge ratio; RT: retention time; VIP; variable importance in the projection; LysoPE: lysophosphatidylethanolamine; LysoPA: lysophosphatidic acid; PA: phosphatidic acid; LysoPC: lysophosphatidylcholine; SM: sphingomyelin.

## Plasma Metabolomics

PCA and PLS-DA models were conducted to visualize the metabolic differences between the two groups (Supplementary Figure 1A-D). PLS-DA plots (Supplementary Figure 1C and 1D) separated metabolites of the two groups clearly, with a R^2^ and Q^2^ (cum) of 0.934 and 0.563 in ESI+, 0.911 and 0.576 in ESI- respectively. Model validity and potential over-fitting of the PLS-DA were checked by performing two hundred permutation tests (Supplementary Figure 1E and 1F). As expected, the R^2^ and Q^2^ values were both lower than the original ones in positive and negative modes.

Finally, a total of 39 metabolites were identified according to the VIP value (VIP > 1.0) based on the PLS-DA model, significance test (*P *< 0.05) and FC (ratio >2 or <1/2) (Supplementary Table 1).
